# Rare and common genetic determinants of metabolic individuality and their effects on human health

**DOI:** 10.1038/s41591-022-02046-0

**Published:** 2022-11-10

**Authors:** Praveen Surendran, Isobel D. Stewart, Victoria P. W. Au Yeung, Maik Pietzner, Johannes Raffler, Maria A. Wörheide, Chen Li, Rebecca F. Smith, Laura B. L. Wittemans, Lorenzo Bomba, Cristina Menni, Jonas Zierer, Niccolò Rossi, Patricia A. Sheridan, Nicholas A. Watkins, Massimo Mangino, Pirro G. Hysi, Emanuele Di Angelantonio, Mario Falchi, Tim D. Spector, Nicole Soranzo, Gregory A. Michelotti, Wiebke Arlt, Luca A. Lotta, Spiros Denaxas, Harry Hemingway, Eric R. Gamazon, Joanna M. M. Howson, Angela M. Wood, John Danesh, Nicholas J. Wareham, Gabi Kastenmüller, Eric B. Fauman, Karsten Suhre, Adam S. Butterworth, Claudia Langenberg

**Affiliations:** 1 British Heart Foundation Cardiovascular Epidemiology Unit, Department of Public Health and Primary Care, University of Cambridge, Cambridge, UK; 2 British Heart Foundation Centre of Research Excellence, School of Clinical Medicine, Addenbrooke’s Hospital, University of Cambridge, Cambridge, UK; 3 Health Data Research UK Cambridge, Wellcome Genome Campus and University of Cambridge, Hinxton, UK; 4 Rutherford Fund Fellow, Department of Public Health and Primary Care, University of Cambridge, Cambridge, UK; 5 MRC Epidemiology Unit, University of Cambridge, Cambridge, UK; 6 Computational Medicine, Berlin Institute of Health at Charité–Universitätsmedizin Berlin, Berlin, Germany; 7 Institute of Computational Biology, Helmholtz Zentrum München–German Research Center for Environmental Health, Neuherberg, Germany; 8 Digital Medicine, University Hospital of Augsburg, Augsburg, Germany; 9 Big Data Institute, University of Oxford, Oxford, UK; 10 Nuffield Department of Women’s and Reproductive Health, University of Oxford, Oxford, UK; 11 Department of Human Genetics, Wellcome Sanger Institute, Wellcome Genome Campus, Hinxton, UK; 12 Open Targets, Wellcome Genome Campus, Hinxton, UK; 13 Department of Twin Research & Genetic Epidemiology, King’s College London, London, UK; 14 Metabolon, Morrisville, NC USA; 15 NHS Blood and Transplant, Cambridge Biomedical Campus, Cambridge, UK; 16 NIHR Biomedical Research Centre at Guy’s and St Thomas’ Foundation Trust, London, UK; 17 NIHR Blood and Transplant Research Unit in Donor Health and Genomics, University of Cambridge, Cambridge, UK; 18 Health Data Science Research Centre, Human Technopole, Milan, Italy; 19 Department of Haematology, University of Cambridge, Cambridge, UK; 20 Institute of Metabolism and Systems Research, University of Birmingham, Birmingham, UK; 21 NIHR Birmingham Biomedical Research Centre, University Hospitals Birmingham NHS Foundation Trust and University of Birmingham, Birmingham, UK; 22 Institute of Health Informatics, University College London, London, UK; 23 Health Data Research UK, London, UK; 24 British Heart Foundation Data Science Centre, London, UK; 25 Vanderbilt Genetics Institute, Vanderbilt University Medical Center, Nashville, TN USA; 26 Clare Hall & MRC Epidemiology Unit, University of Cambridge, Cambridge, UK; 27 Department of Genetics, Novo Nordisk Research Centre Oxford, Oxford, UK; 28 MRC Biostatistics Unit, Cambridge Institute of Public Health, University of Cambridge, Cambridge, UK; 29 The Alan Turing Institute, London, UK; 30 Internal Medicine Research Unit, Pfizer Worldwide Research, Development and Medical, Cambridge, MA USA; 31 Department of Biophysics and Physiology, Weill Cornell Medicine–Qatar, Doha, Qatar; 32 Precision Healthcare University Research Institute, Queen Mary University of London, London, UK

**Keywords:** Quantitative trait, Population genetics

## Abstract

Garrod’s concept of ‘chemical individuality’ has contributed to comprehension of the molecular origins of human diseases. Untargeted high-throughput metabolomic technologies provide an in-depth snapshot of human metabolism at scale. We studied the genetic architecture of the human plasma metabolome using 913 metabolites assayed in 19,994 individuals and identified 2,599 variant–metabolite associations (*P* < 1.25 × 10^−11^) within 330 genomic regions, with rare variants (minor allele frequency ≤ 1%) explaining 9.4% of associations. Jointly modeling metabolites in each region, we identified 423 regional, co-regulated, variant–metabolite clusters called genetically influenced metabotypes. We assigned causal genes for 62.4% of these genetically influenced metabotypes, providing new insights into fundamental metabolite physiology and clinical relevance, including metabolite-guided discovery of potential adverse drug effects (*DPYD* and *SRD5A2*). We show strong enrichment of inborn errors of metabolism-causing genes, with examples of metabolite associations and clinical phenotypes of non-pathogenic variant carriers matching characteristics of the inborn errors of metabolism. Systematic, phenotypic follow-up of metabolite-specific genetic scores revealed multiple potential etiological relationships.

## Abstract (web summary)

Analyses of the genetic architecture of the human plasma metabolome in two large population-based cohorts identify associations between genetically determined metabolite levels and health.

## Main

The plasma metabolome refers to the complete set of circulating metabolites and provides a snapshot of human physiology and a person’s ‘chemical individuality’. The human metabolome is strongly influenced by a variety of endogenous and exogenous factors, including genetic as well as dietary-, drug- and disease-related influences. A range of high-throughput technologies now enable examination of the genetic regulation of biochemical individuality at the population scale. Existing targeted and untargeted platforms provide highly synergistic information due to limited overlap in their coverage of the metabolome^1^. Very large-scale (*N*_max_ ≈ 120,000) genetic studies exist for targeted platforms (up to 168 metabolites) using nuclear magnetic resonance (NMR)^2,3^, but only a few, smaller-scale studies (*N*_max_ ≈ 8,000) have been conducted using the much broader metabolite coverage of untargeted methods (up to 644 metabolites), which have each reported fewer than 150 loci^4,5^. In this Article we present a systematic investigation of the genetic architecture of over 900 metabolites in almost 20,000 men and women. We perform exact conditional analyses, examine allelic heterogeneity and identify genetic co-regulation of multiple metabolites by investigating shared genetic influences on sets of regionally associated metabolites from across a broad array of pathways. Based on the identified genetic associations and manual literature-based curation, we define high-confidence causal genes regulating these metabolites and systematically examine their clinical relevance across over 1,400 phenotypes.

## Results

### Discovery and fine-mapping for individual metabolites

**Fig. 1:**
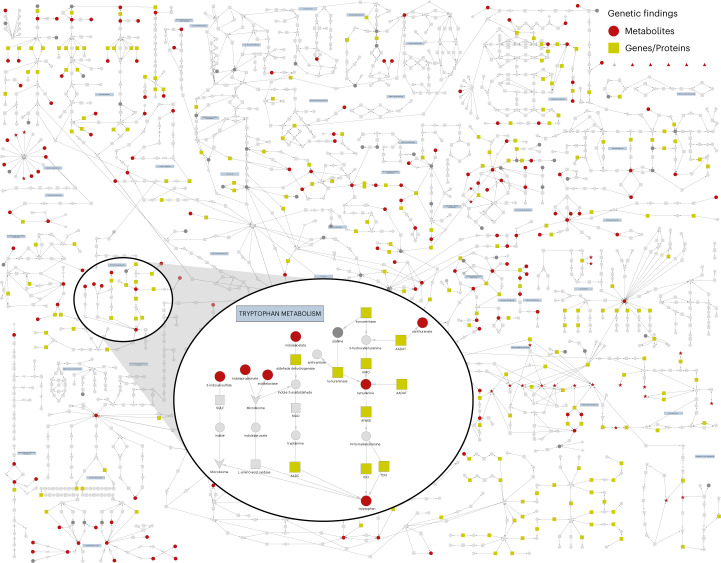
An established map of metabolic pathways. Map of metabolic pathways highlighting 204 of the 632 annotated metabolites analyzed in this study (dark gray and red circles), including 154 with genetic associations (red circles). We also mapped 51 metabolites to class nodes (indicated by star symbols). Of the 46 class nodes, 22 are red, indicating that they contain at least one metabolite with a genetic association. Genes (grey and lime green squares) and causal genes regulating associations discovered in the study (lime green squares; as explained in the section ‘Identification of genetically influenced metabotypes’) are illustrated. Downward-pointing arrowheads indicate a process and upward-pointing triangles indicate a source. The inset focuses on the tryptophan metabolism pathway. An interactive version is available on the accompanying webserver at https://omicscience.org/apps/mgwas.

**Extended Data Fig. 1:**
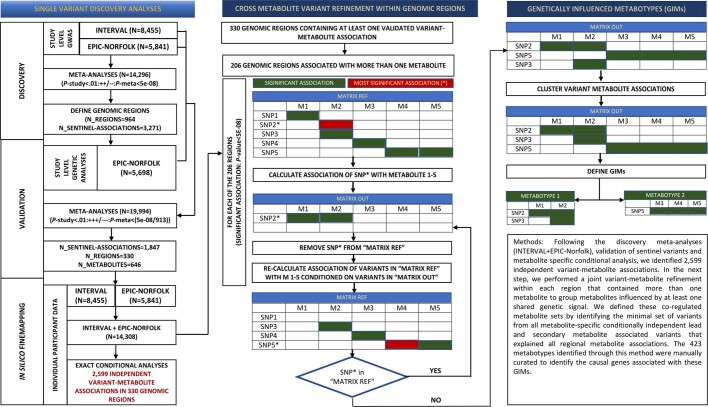
Study design and method of defining genetically influenced metabotypes. Following the discovery meta-analyses (INTERVAL + EPIC-Norfolk), validation of sentinel variants and metabolite specific conditional analysis, we identified 2,599 independent variant-metabolite associations. In the next step, we performed a joint variant-metabolite refinement within each region that contained more than one metabolite to group metabolites influenced by at least one shared genetic signal. We defined these co-regulated metabolite sets by identifying the minimal set of variants from all metabolite-specific conditionally independent lead and secondary metabolite associated variants that explained all regional metabolite associations. The 422 metabotypes identified through this method were manually curated to identify the causal genes associated with these GIMs.

We quantified plasma levels of 913 metabolites for 14,296 individuals of European ancestry from two cohort studies (INTERVAL^6^ and EPIC-Norfolk^7^) using an untargeted mass spectrometry-based platform (Metabolon HD4), as previously described^8^ (Supplementary Table 1). Metabolites with annotated identities were classified into eight broad classes relating to the metabolism of lipids (33.0%), amino acids (16.8%), xenobiotics (10.1%), nucleotides (2.5%), peptides (2.2%), carbohydrates (2.1%), cofactors and vitamins (1.9%) and ‘energy’ (0.8%); additional compounds had an unannotated but unique chemical identity (30.8%) (Supplementary Table 2). In a two-stage genome-wide association meta-analysis (including validation in an additional 5,698 participants from the EPIC-Norfolk study; Extended Data Fig. 1), we identified 1,847 associations of 330 genomic regions with 646 metabolites (Supplementary Table 3). Conditional analysis of these regional associations identified 2,599 conditionally independent variant associations (*P* < 1.25 × 10^−11^; Methods and Supplementary Table 4). We mapped annotated metabolites to 48 established metabolic pathways (Fig. 1). Additionally, we inferred a data-driven metabolic network (Methods) to include metabolites and genetic associations identified, but not covered, by current pathway representations. Both networks (along with details for all association results) can be explored on our webserver at https://omicscience.org/apps/mgwas.

**Fig. 2:**
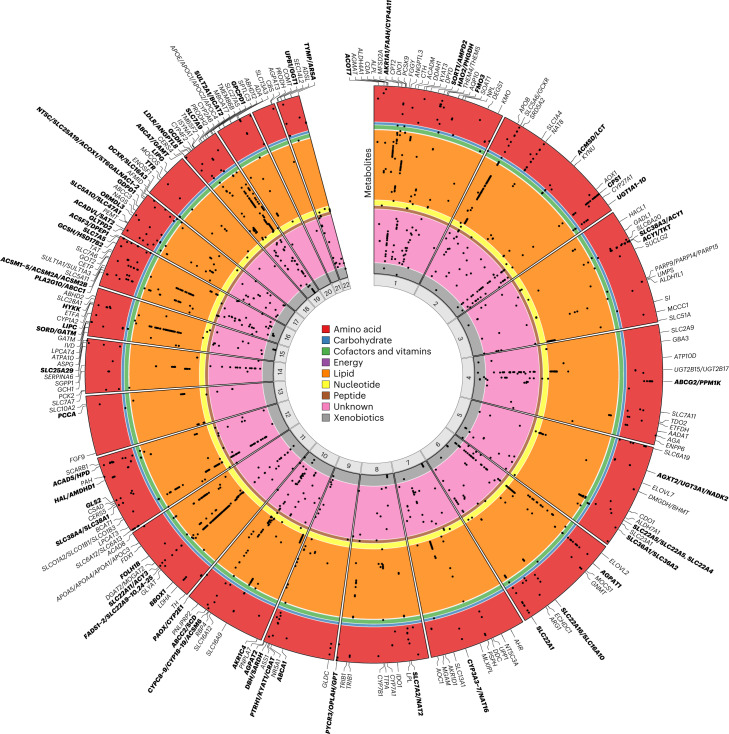
Circular plot illustrating the genomic location of regional associations with metabolites. Metabolites occupy circular bands, within colored sections for each of the assigned metabolic classes: amino acid (*n* = 124), carbohydrate (*n* = 10), cofactors and vitamins (*n* = 15), energy (*n* = 2), lipid (*n* = 241), nucleotide (*n* = 19), peptide (*n* = 12), unannotated compounds (*n* = 185) and xenobiotics (*n* = 38). Metabolite-region associations are indicated by black points. All 646 metabolites with associations are shown. Causal genes are labeled; those in bold indicate regions with more than one GIM (as explained in the section ‘Identification of genetically influenced metabotypes’).

The majority (*n* = 206; 62.4%) of genomic regions associated with multiple metabolites (Fig. 2; https://omicscience.org/apps/mgwas), including half (*n* = 165) with multiple annotated metabolites, specifically 83 (25.2%) associated only with metabolites from within the same class and 82 (24.8%) associated with metabolites from across classes. The *FADS1*/*FADS2* locus associated with the most annotated metabolites (94 lipids), but extensive pleiotropy was also evident for many other regions, including within-class pleiotropy (*PCSK9* and *MFSD2A*) and across-class pleiotropy (*AGPAT1*, *ABCG2*/*PPM1K*, *GCKR*, *SLC22A1* and *ABCC1*/*PLA2G10*).

**Fig. 3:**
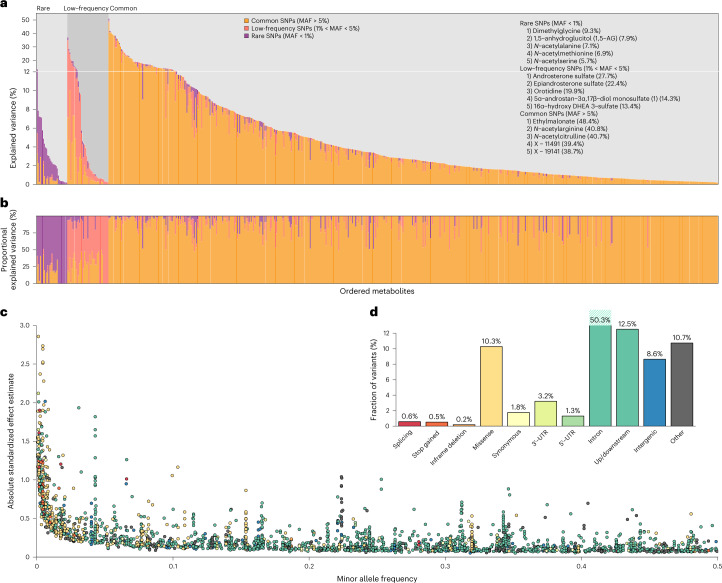
Variance explained, MAF versus effect size and functional annotation. **a**, The percentage of phenotypic variance of each metabolite explained by conditionally independent associations. The variance explained is partitioned into that explained by variants within each MAF bin, and indicated by color: rare (purple), low-frequency (pink) and common (orange). Three groups of metabolites are defined, with rare, low-frequency or common variants explaining the greatest percentage of phenotypic variance of the metabolite. The five metabolites with the greatest percentage of phenotypic variance explained by rare, low-frequency or common variants are listed, with the total percentage of variance explained by all variants in that MAF bin shown in parentheses. **b**, The phenotypic variance of each metabolite explained by variants within each MAF bin as a percentage of the variance explained by all conditionally independent associations. **c**, MAF versus association effect size for conditionally independent associations, with variants colored by functional annotation class as indicated in **d**. **d**, A bar plot of the frequency of variants in each functional class.

The phenotypic variance explained by conditionally independent variants ranged from 0.2% to 51% (mean 5.2%) (Fig. 3a,b and Supplementary Table 5). The mean was highest for amino acid (6.36%; *n* = 124) and energy (7.36%; *n* = 2) classes and lower for peptide (2.65%; *n* = 12), carbohydrate (2.69%; *n* = 10) and xenobiotic (3.41%; *n* = 38) classes. The range in variance explained suggests different genetic architectures both between and within classes. For annotated metabolites (*n* = 461) and unannotated compounds (*n* = 185) with at least one association, the mean variance explained (5.08% and 5.65%, respectively) and the mean number of associated variants (4.05 (range 1–16) and 3.95 (range 1–16), respectively) were similar. For common (minor allele frequency (MAF) > 5%), low-frequency (1% < MAF ≤ 5%) and rare (MAF ≤ 1%) variants, the maximum variances explained for any single metabolite were 48.4%, 27.7% and 9.3%, respectively (Fig. 3a,b and Supplementary Table 5).

**Extended Data Fig. 2:**
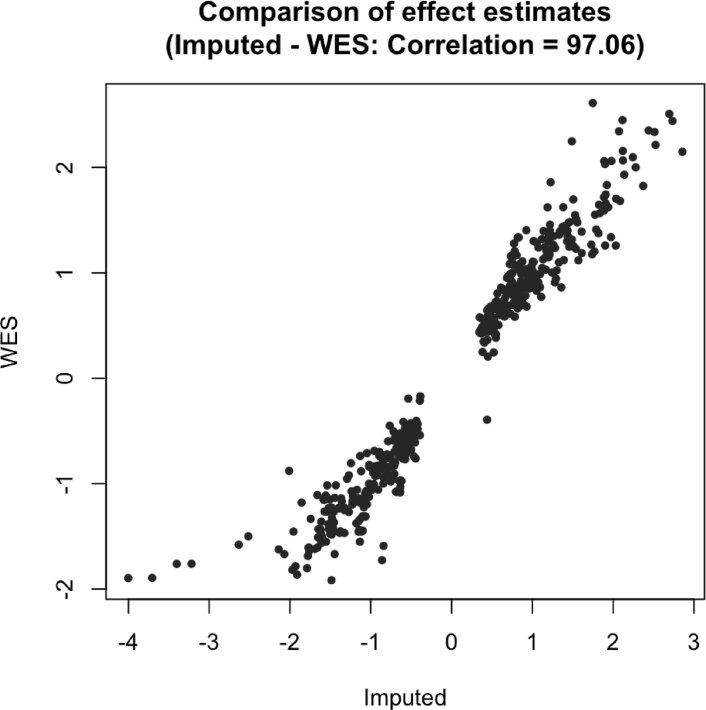
Comparison of rare variant effect sizes with WES results. Comparison of rare variant effect sizes between the discovery meta-analysis, and the WES analysis in a subset of 3,924 samples from the INTERVAL study (R^2^ = 98.33). 122 (46.2%) of all rare variant associations were testable using WES analysis. All 122 were directionally consistent and 118 were at least nominally significant (P-value < 0.05).

Functional annotation using Ensembl Variant Effect Predictor (VEP) indicated that 177 (11.6%) of the conditionally independent variants had a direct functional consequence on the transcript (Fig. 3d). In total, 692 (26.6%) conditionally independent associations had large absolute effect sizes (*β*) (>0.3 s.d. per allele), 439 (63.4%) of these with low-frequency or rare variants (Fig. 3c). Overall, 245 (9.4%) associations were with rare variants, which accounted for 152 (9.9%) of conditionally independent variants. We used whole exome sequence (WES) data from a subset of INTERVAL participants^9^ for technical validation of rare variant associations and found a strong correlation of effect sizes (*R*^2^ = 0.98; Extended Data Fig. 2), confirming that the associations were not genotyping or imputation artefacts.

Of the 330 associated genomic regions, 225 were not reported by the previous largest genetic studies using the Metabolon assay^4,5^. For overlapping metabolites, we replicated 302 (83.2%) reported region–metabolite associations (involving 106 genomic regions and 226 metabolites; associations with either the reported variant or the variant in linkage disequilibrium (LD), *r*^2^ > 0.1) at *P* < 5 × 10^−8^ (Methods). For those metabolites, our conditional analyses identified a further 212 conditionally independent variant–metabolite associations independent of the previously reported associations (Supplementary Table 6). In addition, within previously reported regions we identified associations with an additional 424 metabolites (1,046 conditionally independent variant–metabolite associations), demonstrating the value of both larger sample size and broader quantification of metabolites for identifying genetic determinants of metabolite variation.

### Identification of genetically influenced metabotypes

**Fig. 4:**
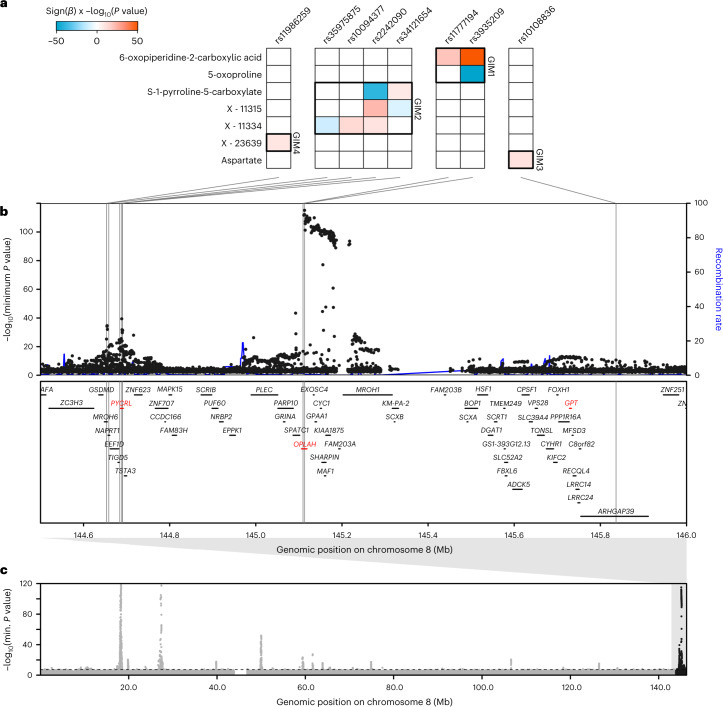
Example of defining GIMs within a genomic region. At a 2.55-Mb region on chromosome 8 (region 512), metabolite associations fall into four sets (GIMs) acting through three genes (*PYCR3*, *OPLAH* or *GPT*) with known roles in metabolism. **a**, Four GIMs defined by overlap in the genetic regulation of metabolite sets. Matrices display the −log_10_(*P*) (capped at 50) and direction of effect (higher, red; lower, blue) for associations from stepwise conditional models, fitting the variants in the following order: rs3935209, rs2242090, rs11777194, rs10094377, rs35975875, rs10108836, rs11986259, rs34121654. GIM 1: two variants associating with 6-oxopiperidine-2-carboxylic acid and 5-oxoproline; the causal gene is *OPLAH*, encoding 5-oxoprolinase, which catalyzes the ATP-dependent hydrolysis of 5-oxoproline to glutamic acid (5-oxoproline and the structurally closely related 6-oxopiperidine-2-carboxylic acid associated in this cluster). GIM 2: four variants associating with S-1-pyrroline-5-carboxylate and the unannotated metabolites X-11315 and X-11334; the causal gene is *PYCR3*, a pyrroline-5-carboxylate reductase that generates proline from S-1-pyrroline-5-carboxylate (the strongest associated metabolite in this cluster). GIM 3: a single variant associating with aspartate; the causal gene is *GPT*, encoding alanine aminotransferase, which takes alanine as a substrate and produces glutamate, which is one step removed from the associated metabolite aspartate. GIM 4: a single variant associating with the unannotated metabolite X-23639. **b**, Regional association indicating genomic positions of the associated variants (black lines) and causal genes (in red). **c**, Manhattan plot of chromosome eight, with the *y* axis capped at 120 for clarity. All *P* values presented were derived from linear mixed models.

Within genomic regions, we grouped metabolites influenced by at least one shared genetic signal into genetically influenced metabotypes (GIMs)^10^. We defined these co-regulated metabolite sets by identifying the minimal set of variants from all metabolite-specific conditionally independent lead- and secondary metabolite-associated variants that explained all regional metabolite associations (Extended Data Fig. 1). To illustrate, one 2.55-Mb region on chromosome 8 showed associations between eight variants and seven metabolites, which were partitioned into four distinct GIMs (Fig. 4; https://omicscience.org/apps/mgwas). We identified 423 GIMs, which included up to 15 lead genetic variants (median = 1) and up to 89 metabolites (median = 2). For 264 (62.4%) GIMS, we assigned one of 253 likely causal genes (or gene sets) by extensively mining the biochemical literature (Methods and Supplementary Table 7).

### Biological insights from GIMs

GIMs can provide insights into the diverse ways in which genetic variation influences metabolism and chemical individuality. We identify examples of GIMs with important clinical implications (for example, *SRD5A2* and *DPYD*), providing insights into fundamental metabolite physiology, indicating different roles of a multi-functional protein (*TTR*, *SLC7A2* and *SLC7A5*), and with tissue-specific effects through the same protein (for example, *CPS1*).

**Fig. 5:**
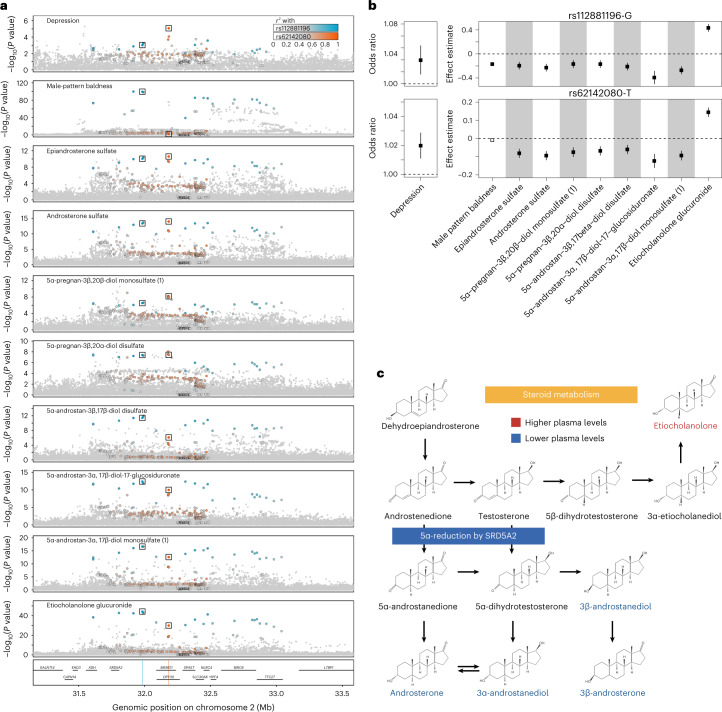
Clinical implications of genetic variation at the *SRD5A2* locus. **a**, Stacked regional association plots for eight steroid metabolites, the risk of male-pattern baldness and depression in a 2-Mb window around the most likely causal gene, *SRD5A2*. Association statistics (*P* values from linear mixed models) for levels of plasma metabolites were derived from linear regression models as described in the text, and summary statistics for male-pattern baldness and depression were extracted from the literature^17,19^. The two-color gradients indicate the LD (*r*^2^) with the candidate causal variants identified using multi-trait colocalization: rs112881196 (blue, lead signal for male-pattern baldness) and rs62142080 (orange, lead signal for depression). **b**, Forest plot showing effect estimates (box) and 95% confidence intervals for rs112881196 (top panel) and rs62142080 (lower panel) across all traits considered. Effects for depression are given as odds ratios, because logistic regression models were used for association testing, whereas effects for all other traits were estimated using linear regression models. Effect estimates and corresponding standard errors for male-pattern baldness and depression were obtained from the same studies as described in the text. Sample sizes for metabolites are described in Supplementary Table 8. Open symbols indicate non-significant effects (*P* > 0.05). **c**, Scheme describing the putative mechanism by which the two genetic variants nearby *SRD5A2* alter steroid metabolism. Lower plasma levels of metabolites downstream of 5α-reduction of androgenic steroids but higher levels of the main 5β-reduced androgen metabolite etiocholanolone indicate lower activity of steroid 5α-reductase 2 (SRD5A2) conferred by variants associated with a lower risk for male-pattern baldness (via rs112881196) but increased risk for depression (via rs62142080). Parts of this figure were created with BioRender.com.

We identified variation near *SRD5A2*, the gene product being a target of antiandrogen drugs for the treatment of male-pattern baldness and benign prostatic hyperplasia^11^, as associated with eight steroid metabolites of steroid hormone biosynthesis, including six androgen metabolites (Fig. 5 and Supplementary Table 7). *SRD5A2* encodes steroid 5α-reductase 2 (SRD5A2), which activates testosterone to dihydrotestosterone, the most potent ligand for the nuclear androgen receptor; SRD5A2 is also involved in the inactivation of gluco- and mineralocorticoids^12,13^. We observed genetic associations consistent with lower SRD5A2 activity, with lower levels of conjugates of androsterone, epiandrosterone, 3α-androstanediol and 3β-androstanediol (that is, metabolites downstream of 5α-reduction of androgenic steroids), but higher levels of the major 5β-reduced androgen metabolite etiocholanolone (Fig. 5). Specifically, lower levels of androsterone sulfate and epiandrosterone sulfate have been reported to indicate reduced SRD5A2 activity^14^, and inhibitors of SRD5A2, such as finasteride, are widely used to treat enlarged prostate and male-pattern hair loss^11^. Although direct evidence of causality is currently lacking, depression and suicidality have been reported by antiandrogen users^15,16^, and manufacturers are required to list these as potential adverse effects in some countries. Variants in the *SRD5A2* region have been previously associated with the risk of male-pattern baldness^17^. We performed colocalization using HyPrColoc^18^ and identified a shared genetic signal between multiple androgen metabolites and male-pattern baldness (posterior probability for a shared causal variant across all phenotypes (PP) = 0.97), with rs112881196 being a potential driver of this signal. This variant is 176 kb upstream of *SRD5A2*, in strong LD (*r*^2^ > 0.9) with the strongest genome-wide association analysis (GWAS) lead variant at this locus, and showed directionally consistent associations indicating greater SRD5A2 activity and risk of male-pattern hair loss. We identified a separate, shared genetic association between androsterone sulfate, epiandrosterone sulfate and depression^19^ (PP = 0.98) (Methods), with rs62142080 the most likely causal variant. In line with the increased risk of depression reported in antiandrogen drug users, the major allele (T) of rs62142080 was associated with lower metabolite levels and a higher risk of depression^19^ (*P* = 9.36 × 10^−6^; Fig. 5), supporting concerns about widespread use of SRD5A inhibitors^15,16^.

Another clinically relevant example is related to *DPYD*, encoding dihydropyrimidine dehydrogenase, an enzyme involved in the breakdown of pyrimidines such as uracil and thymine. Variants that reduce *DPYD* activity can limit the breakdown of commonly used fluoropyrimidine cancer chemotherapies, such as 5-fluorouracil and capecitabine, causing severe or life-threatening toxicity in 10–40% of patients treated^20^. Several variants near *DPYD* are routinely used to identify patients who should be started on a reduced chemotherapy dose, but an estimated 70–80% of early-onset life-threatening 5-fluorouracil toxicities are not adequately identified by current screening panels^20,21^. We identified four variants in the *DPYD* region at which minor alleles were specifically associated with higher plasma uracil levels (Supplementary Table 7), including two rare variants currently recommended for pre-treatment screening^20^ (rs3918290: MAF 0.5%, *β*_marginal_ = 1.243, *P*_marginal_ = 1.49 × 10^−38^; rs67376798: MAF 0.8%, *β*_marginal_ = 0.768, *P*_marginal_ = 2.23 × 10^−25^) (Supplementary Table 8). We also identified two common variants with more modest effect sizes (rs60392383: MAF 20.6%, *β*_marginal_ = 0.111, *P*_marginal_ = 8.16 × 10^−12^; rs72977723: MAF 12.1%, *β*_marginal_ = 0.308, *P*_marginal_ = 2.03 × 10^−54^) (Supplementary Tables 7 and 8). The variant rs72977723 tags the toxicity-associated ‘HapB3’ haplotype, which is included in screening recommendations by measuring rs56038477 (ref. ^20^). Although we found rs56038477 to be associated with uracil in single-variant analyses (*P*_marginal_ = 1.06 × 10^−11^), this association was almost completely attenuated in a joint statistical model with rs72977723 (rs56038477: *P*_joint_ = 0.342; rs72977723: *P*_joint_ = 3.11 × 10^−45^; Supplementary Table 9), suggesting that rs72977723 better captures the effects of the HapB3 haplotype on uracil breakdown. We found that a substantial fraction (17.8%) of our participants carry the minor allele of rs72977723 but do not carry other alleles used for screening, suggesting that the addition of rs72977723 to screening panels could identify a substantial number of additional individuals who are at risk of treatment-induced toxicity.

Distinct GIMs that share the same causal gene can highlight different functions of the same gene product, such as for multi-functional transporters. *TTR* encodes transthyretin (TTR), which is involved in the transport of both the thyroid hormone thyroxine and retinol (by forming a complex with the retinol binding protein, RBP)^22^. We found two GIMs that included variants probably affecting TTR function differently (Supplementary Table 7). The first GIM was represented by a rare variant (rs184097503) in perfect LD with rs28933981 (p.T119M), which is known to enhance the stability of TTR and leads to higher plasma TTR levels and greater thyroxine transport capacity^23^. In line with this, we found a strong association of the minor allele (C) with higher thyroxine levels (*P* = 1.14 × 10^−12^) but no association with retinol (*P* = 0.573) levels (Supplementary Table 8). The second GIM was represented by a common variant (rs1667237) at which the minor allele (C) was strongly associated with higher retinol (*P* = 1.72 × 10^−14^) levels. Although this variant was only modestly associated with higher thyroxine levels in our study (*P* = 0.003), a strong proxy (rs1080094, *r*^2^ = 0.98) has been robustly associated with circulating free thyroxine, that is, the non-protein-bound fraction, in a study of ~50,000 participants^24^. Although thyroxine has several transporters, retinol is exclusively transported by the TTR–RBP complex, suggesting that this lack of redundancy for retinol transport could explain the stronger association with plasma retinol levels seen for this GIM.

Other examples of GIMs capturing multiple functions of a gene include those of the membrane solute transporters, *SLC7A2*, distinct variants being associated with either lysine or arginine levels, and *SLC7A5*, distinct variants being associated with either kynurenine or imidazole lactate levels (Supplementary Table 7).

We observed tissue segregation of GIMs mapping to the same causal gene. For example, two GIMs at *CPS1* harbor associations with either glycine-related metabolites (rs1047891) or citrulline (rs13411696 and rs114764732) (Supplementary Table 7). *CPS1* encodes carbamoyl phosphate synthetase, a key liver and small-intestine enzyme regulating entry into the urea cycle. Disease-causing mutations have been implicated in the allosteric *N*-acetyl-l-glutamate-binding domain^25^, and the missense variant rs1047891 (p.T1405N) potentially causes an amino-acid change in the *N*-acetyl-l-glutamate-binding domain, influencing enzyme activation and thereby restricting flux into the urea cycle (primarily in the liver), with consequential effects on glycine metabolism. This is a known association with an established much stronger effect in women and a causal role in coronary disease^26^. In the small intestine, which lacks the full complement of urea-cycle enzymes, *CPS1* contributes to the generation of citrulline, a metabolite used as a clinical biomarker of intestinal function and enterocyte mass^27^. Thus, the citrulline-associated GIM may reflect a tissue-specific effect of altered *CPS1* expression. We observed a shared signal between the citrulline GIM and *CPS1* expression (using HyPrColoc; PP > 0.8) for 10 of the 49 GTEx (V8) tissues^28^, although not in tissues known for high *CPS1* expression.

### Genes known to cause IEMs

IEMs are metabolic diseases caused by rare genetic variants that lead to metabolite deficiency and/or accumulation, with severe phenotypic consequences if left undetected or untreated^29,30^. Many of the identified associations with metabolite levels in this population-based study are in or near genes known to cause IEMs, as has previously been reported for *PCSK9*, *LPL* and *CPS1* (refs. ^1,31^). We identified an eightfold enrichment of genes known to cause IEMs among the causal genes (Methods; fold enrichment of 8.10, *P* = 7.88 × 10^−57^). After accounting for overlapping signals across detected GIMs, 88 (27.50%) regions harbored at least one of 97 IEM genes (Supplementary Table 10). Within these regions, we identified 14 known or likely pathogenic IEM variants (as annotated within ClinVar^32^, for the variant or proxies in LD (*r*^2^ > 0.6 or *D*′ > 0.9); Supplementary Table 11). These variants (MAF 0.09–7.90%) had associations with an absolute *β* of 0.526–1.97 per 1 s.d. difference in metabolite levels per allele, and mapped to genes known to cause amino-acid disorders, fatty-acid-oxidation disorders and mitochondrial disorders. In addition, we identified 185 variants without established pathogenicity in ClinVar (MAF 0.09–49.54%, having associations with absolute *β* of 0.0628–2.75 per 1 s.d. difference in metabolite levels per allele) that had primary or secondary IEM-specific metabolite consequences, that is, were most strongly associated with a metabolite that was identical or closely related to those affected in the corresponding IEM (Supplementary Table 12).

**Extended Data Fig. 3:**
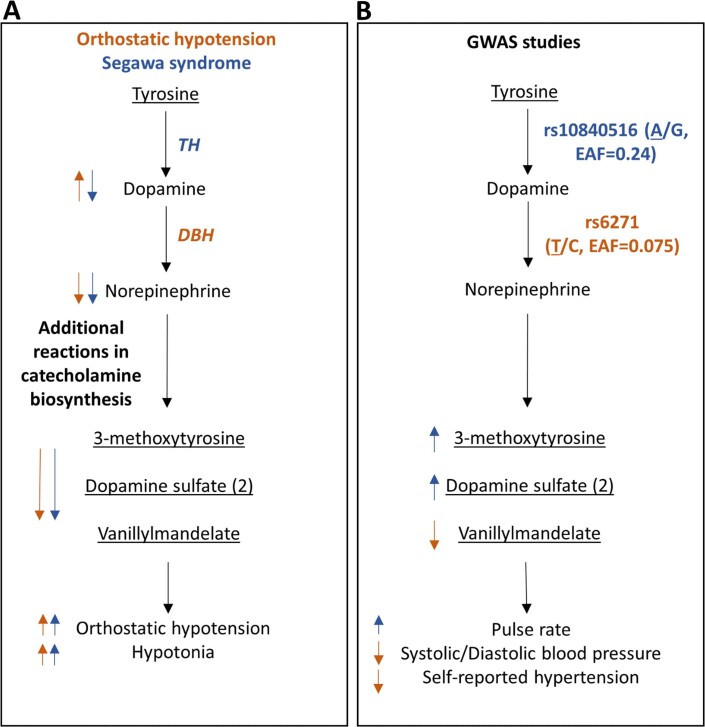
Common variants at IEM genes have metabolic and phenotypic consequences mimicking those observed in corresponding IEM. **a**) Rare mutations at the *DBH* and *TH* genes are known to cause the IEMs orthostatic hypotension (OMIM #223360, coloured in orange) and Segawa syndrome (OMIM #605407, coloured in blue). **b**) In this study, we found common variants at these genes that are associated with metabolic and phenotypic consequences mimicking those observed in the corresponding IEMs.

**Extended Data Fig. 4:**
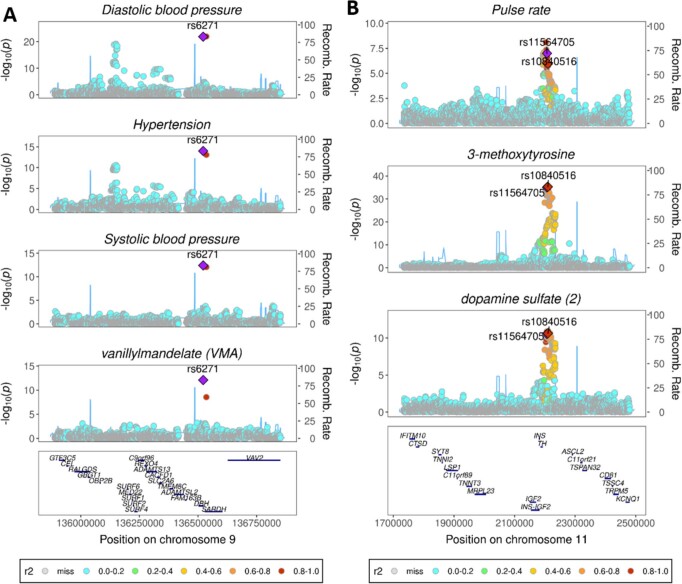
Colocalisation of metabolic and phenotypic associations at *DBH* and *TH*. **a**) At GIM547.3, the *DBH* variant rs6271 is a strong likely-causal candidate variant for shared signals between decreased plasma vanillylmandelate levels, decreases in automated readings of systolic and diastolic blood pressure (N = 436,424), and a decrease in self-reported hypertension in UK Biobank (N = 462,933). **b**) At GIM604.1, the *TH* variant rs11564705 (MAF = 24%, r^2^ = 0.98 with the variant rs10840516 identified in this study) is a strong likely-causal candidate variant for shared signals between increased plasma levels of 3-methoxytyrosine and dopamine sulfate (2) and an increase in automated readings of pulse rate in UK Biobank (N = 436,424).

**Extended Data Fig. 5:**
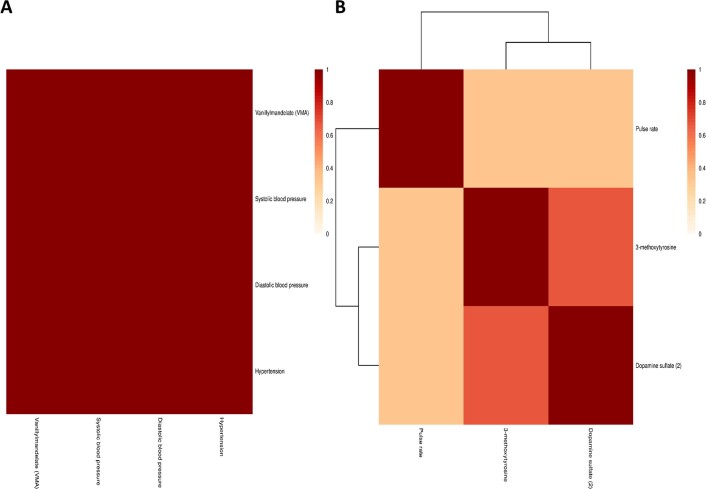
Sensitivity analyses heatmaps for colocalisation analyses at *DBH* and *TH*. Sensitivity analyses heatmaps for colocalisation at **a**) *DBH* and **b**) *TH*. Heatmaps showing the proportion of clusters that traits share across tested configurations of prior2 values (0.99, 0.999) and regional and alignment thresholds (0.6, 0.7, 0.8, 0.9).

We investigated whether carriers of non-pathogenic variation at IEM genes had phenotypic features characteristic of those seen in IEM patients and found evidence for common representation of IEM-related features for several genes. For example, orthostatic hypotension-1 (OMIM #223360) is an autosomal recessive disorder characterized by mutations in dopamine beta hydroxylase (*DBH*), which converts dopamine to norepinephrine. Mutation carriers have higher plasma levels of dopamine and low levels of norepinephrine (noradrenaline) and epinephrine (adrenaline), leading to dysregulation of autonomic functions such as control of temperature, blood pressure and vascular tone^33,34^ (Extended Data Fig. 3). We identified associations of a missense variant in *DBH* (rs6271, p.R549C, MAF = 7.45%) with lower levels of the norepinephrine catabolite vanillylmandelate (*β* per minor (T) allele = −0.164, *P* = 8.00 × 10^−13^), as well as lower systolic and diastolic blood pressure and lower risk of hypertension in independent, non-overlapping studies^35–38^. We found strong evidence of a shared genetic signal for these IEM characteristic features (PP = 0.97), with rs6271 as the likely underlying causal variant in multi-trait colocalization analysis (Extended Data Figs. 4 and 5). We observed similar phenotypic convergence for a common intergenic variant, rs10840516 (MAF = 24%). The likely causal gene, tyrosine hydroxylase (*TH*), catalyzes the conversion of tyrosine to levodopa upstream of the biochemical reaction catalyzed by *DBH*. Mutations at the *TH* gene can lead to dysregulated dopamine metabolism, which in turn may also affect pulse rate and blood pressure regulation (Extended Data Fig. 3)^39^. Variant rs10840516 associated with higher plasma levels of 3-methoxytyrosine, dopamine sulfate and higher pulse rate in the UK Biobank (*β* per minor (A) allele 0.012, *P* = 1.10 × 10^−6^). Multi-trait colocalization provided strong evidence of a shared genetic signal between these traits (PP = 0.79; likely causal variant rs11564705, in high LD (*r*^2^ ≥ 0.97) with rs10840516; Extended Data Figs. 4 and 5).

### GIMs enable variant to function annotation at GWAS loci

**Extended Data Fig. 6:**
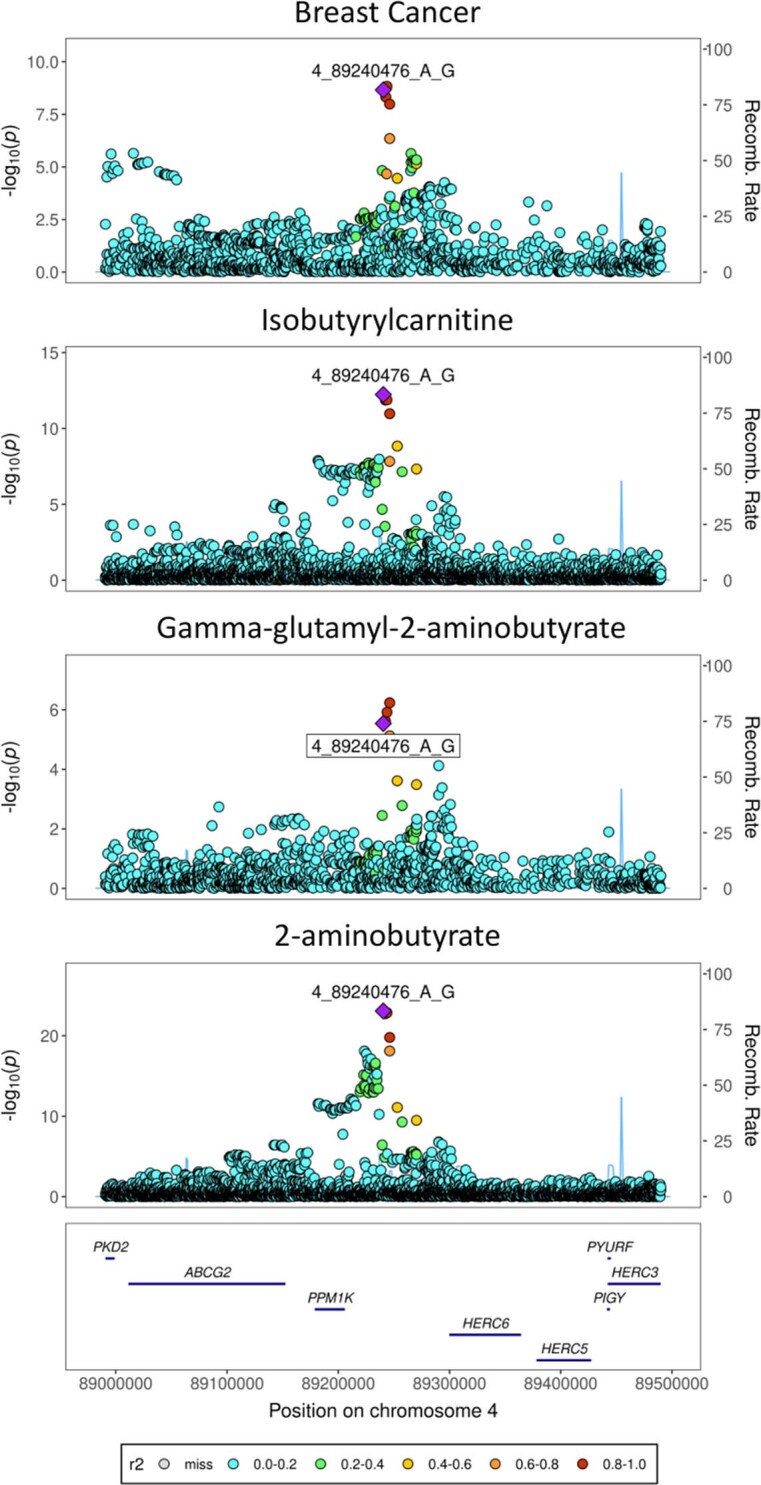
Colocalisation between *PPM1K* and BCAA-catabolites. Stacked regional plots showing colocalization between breast cancer and BCAA-catabolites, 2-aminobutyrate, isobutyrylcarnitine and gamma-glutamyl-2-aminobutyrate colocalise (PP = 0.98) within *PPM1K*.

The high-confidence causal gene assignment for GIMs can guide identification of disease-causing mechanisms at known GWAS loci. We systematically investigated associations of GIM defining variants (or proxies; at *r*^2^ > 0.8) with clinical outcomes using the NHGRI-EBI GWAS Catalog and PhenoScanner^40^ (Supplementary Table 13). Variants within 54 GIMs were associated (*P* < 5 × 10^−8^) with the lead variant for at least one of 41 categories of complex diseases, including coronary artery disease (CAD; 13 GIMs) and chronic kidney disease (CKD, eight GIMs) (Supplementary Table 13). Causal genes for these GIMs included established genes for CAD (for example, *PCSK9*, *SORT1* and *LDLR*), age-related macular degeneration (*LIPC* and *APOE*/*APOC*[1,2,4]), Crohn’s disease (*GCKR* and *FADS2*) and CKD (*GATM*). Causal genes for 15 GIMs are targets of approved drugs or clinical-phase drug candidates^41^ (Supplementary Table 13). We followed up rs17014016 in *PPM1K*, recently reported to be associated with an increased risk of breast cancer^42^. *PPM1K* encodes a phosphatase essential to catabolism of branched-chain amino acids (BCAAs)^43^. We demonstrate that the genetic associations at *PPM1K* with the BCAA catabolites 2-aminobutyrate, isobutyrylcarnitine and gamma-glutamyl-2-aminobutyrate colocalize (PP = 0.98) with the association with breast cancer (Methods and Extended Data Fig. 6), supporting a role for BCAA catabolism in breast cancer etiology^44^.

### From molecules to clinical presentations

**Fig. 6:**
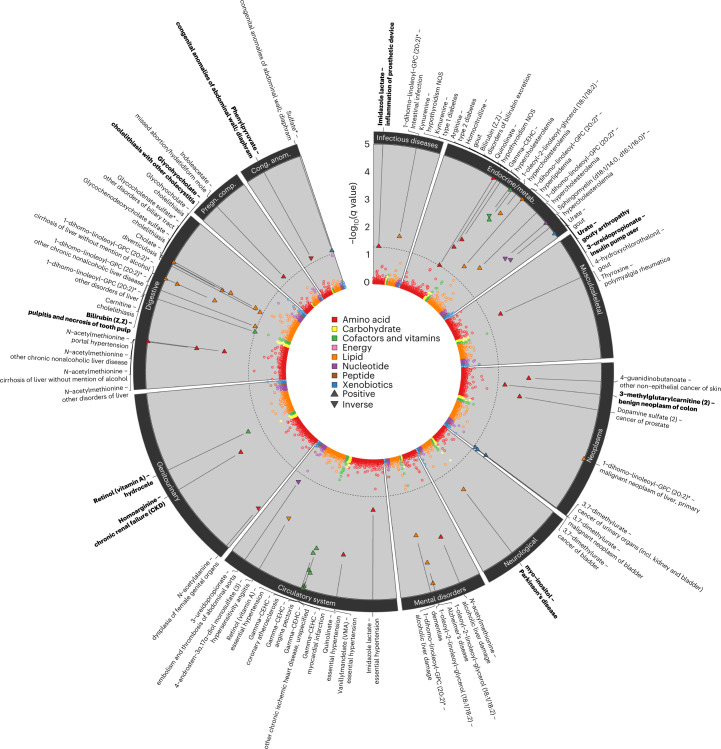
Summary of phenome-wide associations with metabolite scores. The circos plot displays adjusted *P* values (*q* value) from logistic regression models testing for pairwise associations between 155 genetically predicted metabolite levels (scores) and 1,457 phecodes in the UK Biobank. Each dot represents one metabolite–phecode association, and colors reflect metabolite classes. Associations passing the multiple testing correction cutoff (*q* < 0.05) are indicated by larger triangles, the orientation of which indicates the association direction, and are annotated at the outer margins of the plot. Metabolite score–phecode associations with robust evidence for a dose–response relationship are indicated in bold (see text). Effect estimates, standard errors and *P* values are provided in Supplementary Table 14.

To systematically test how genetic variation in metabolite levels is linked to a broad spectrum of diseases, we imputed genetically predicted metabolite levels (‘metabolite scores’) in UK Biobank participants using weighted genetic scores, and estimated their associations with 1,457 collated disease terms (‘phecodes’)^45^ derived from electronic health records (Methods). We considered 155 annotated metabolites with at least two associated, non-pleiotropic, genetic variants. We identified 60 metabolite score–phecode associations at a 5% false discovery rate, involving 33 metabolites and 44 phecodes (Fig. 6 and Supplementary Table 14). Results included well-established links between metabolites and diseases, such as urate and gout (odds ratio (OR) per 1-s.d.-higher metabolite level, 2.22; 95% CI, 2.11–2.35; *P* = 5.9 × 10^−186^), bile acids and cholelithiasis (for example, glycohyocholate: OR, 0.57; 95% CI, 0.51–0.64; *P* = 2.7 × 10^−23^) and complex lipids and hypercholesterolemia (for example, 1-dihomo-linoleoyl-GPC (20:2): OR, 1.84; 95% CI, 1.60–2.21; *P* = 1.4 × 10^−17^).

**Extended Data Fig. 7:**
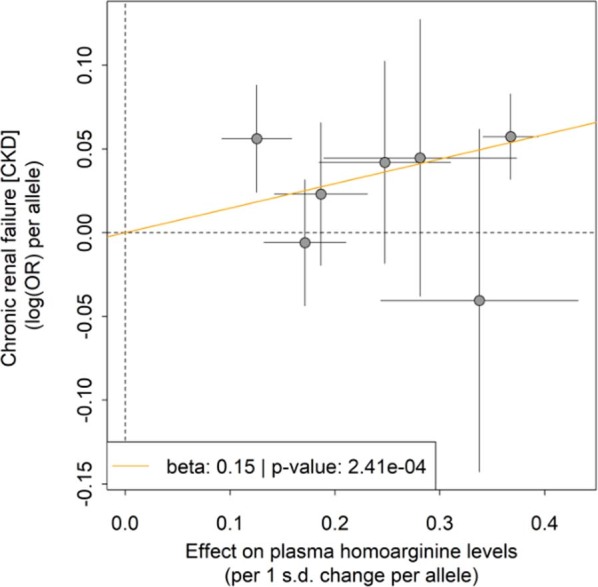
Dosage plot for homoarginine and chronic renal failure variant associations. Dosage plot showing, for each variant in the homoarginine metabolite score, the estimated risk of chronic renal failure (log(OR per allele) versus the estimated effect on homoarginine levels (per 1 s.d. change per allele). Each dot represents the point estimates from the respective linear/logistic regression models using the genetic variant as exposure and either the metabolite or disease status as outcome (n = 334577, n cases=16389; for metabolite, n = 14295 cases for chronic renal failure). Lines indicate 95%-confidence intervals.

For these prioritized associations, we tested for a dose–response relationship using a Mendelian randomization (MR) framework (Methods) and identified 30 pairs with apparent dose–response relationships, with ten providing strong evidence; that is, there were at least three variants in the score and no evidence for between-variant heterogeneity (*P* > 0.05, Methods). These included a positive association between plasma levels of homoarginine and risk of CKD (OR, 1.16; 95% CI, 1.09–1.23; *P* = 6.5 × 10^−7^; Extended Data Fig. 7), which contrasts with observational studies linking higher homoarginine levels with lower renal and cardiometabolic disease risk^46,47^. Our analysis provides an important advance from previous MR analysis using fewer instruments, which yielded null results^48^, highlighting the need to closely monitor kidney function when adopting supplementation strategies with homoarginine^49^ due to the potential adverse effects. Much attention for a possible involvement of arginine-related metabolites in cardiometabolic disease has been paid to either arginine itself^50^ or its possible adverse catabolites, (a)symmetric dimethylarginine (ADMA and SDMA), based on their suggested vasodilatory role^51,52^, with some evidence from single-locus MRs for a putative adverse effect of higher arginine on CAD^53^. Although our metabolomics platform cannot distinguish between ADMA/SDMA, we observed only weak evidence for a possible role of arginine in cardiometabolic and renal disease (for example, diabetic retinopathy, *P* = 6.1 × 10^−4^, or cystic kidney disease, *P* = 1.1 × 10^−3^). The observations that genetic variants associated with homoarginine are probably linked to transporters with specific affinity to homoarginine (*SLC15A19* and *SLC7A7*) and that the known CKD intergenic variant rs1145091 (near *GATM*) was the strongest variant for plasma homoarginine levels argue for a possible distinct role of plasma homoarginine compared to arginine-related metabolites in plasma in CKD pathology. Furthermore, genetically predicted plasma levels of 3-methylglutarylcarnitine were inversely associated with benign neoplasms of the colon (OR, 0.89; 95% CI, 0.85–0.94; *P* = 6.2 × 10^−6^). 3-Methylglutarylcarnitine is a downstream catabolite of leucine metabolism, and elevated plasma levels are used to diagnose 3-hydroxy-3-methylglutaryl-coenzyme A lyase deficiency^54,55^, an IEM characterized by frequent metabolic acidosis with a severe liver phenotype but no reported impact on neoplasms of the colon. The multi-locus nature of our observation points towards a protective role of high 3-methylglutarylcarnitine plasma levels outside of the IEM, an observation that warrants further experimental follow-up to establish possible underlying mechanisms.

## Discussion

Human metabolism and metabolic responses are highly individual and are dysregulated in many common and rare diseases. By conducting the largest genetic study of untargeted metabolomics, we have identified hundreds of genetic variants acting in complex metabolic hotspots in the genome and with large effects on many circulating metabolites. We used this information to define GIMs, which represent the genetic basis of chemical individuality and explain a substantial amount of inter-individual differences in plasma levels of over 600 metabolites. To investigate the consequences of genetic differences in chemical individuality for human health, we pursued a variety of approaches with phenotypic follow-up for a large range of rare and common human conditions. We show convergence of metabolic and phenotypic presentations of genes known to cause rare IEMs with variants at these genes identified in this study of the general population.

Previous studies generally treated metabolites as distinct entities in association analysis^1,4,5,56^, and very few considered the extensive local co-regulation of either biochemically related or seemingly unrelated metabolites (that is, those across different biochemical classes^10^). Our approach to systematically identify such metabolic hotspots in the genome provides a framework that will probably provide additional insights for other domains of molecular traits such as gene or protein expression, but also to disentangle genetic co-regulation in the medical phenome more generally, as exemplified by the multiple signals at *SRD5A2*.

The lack of identification of causal genes remains one of the most important limitations for the successful translation of GWAS findings so far. The intrinsic biochemical link between the function of proteins encoded by genes close to metabolite-associated variants provides direct metabolically informed evidence for causal gene assignment based on decades of biochemical experiments. We have exemplified how this information can identify causal genes for, and provide mechanistic insight into, known loci for diverse diseases, as well as providing examples of genetically predicted metabolite levels robustly associated with complex diseases.

We have previously shown hundreds of associations between plasma metabolite levels and future onset of multiple diseases^8^, and have hypothesized that few of those are likely to be causal, but instead reflect ‘common antecedents’ underlying both metabolite levels and disease risk. The genetic approaches for causal inference used in this study appear to support this notion, as we found few examples with strong genetic support for a causal association between a metabolite and a disease. However, the associations identified here will enable causal assessment for future metabolome-wide association studies across many diseases. This provides a cost-effective and rapid way to (de)prioritize exposures for assessment in randomized trials, to avoid failures, such as has been seen for vitamin C and diabetes^57^ or selenium and prostate cancer^58^. Furthermore, more diverse and large-scale efforts will identify genetic determinants for those metabolites not yet captured here or those for which we have identified only single or non-specific variants.

A third of compounds investigated were unannotated, so future work will include further triangulation of associated variants and causal gene assignment to assist their identification. Our results rely on individuals of European ancestry, and investigation in other ancestries will probably provide additional insights^59,60^. We note that, for certain metabolites, measurement error might have contributed to low estimates of variance explained. Our phenome-wide approach using metabolite scores could be extended at multiple levels: (1) genome-wide scores will probably provide more statistical power, although at the cost of biological specificity, (2) we could use a more refined approach to select metabolite- or pathway-specific genetic instruments to generate metabolite scores and (3) we could extend application to studies with greater numbers of disease cases.

Our results reveal a genomic landscape that accounts for chemical individuality, with important and potentially actionable insights for human health. Future integration with molecular layers providing complementary information, such as protein or gene expression, and obtained in diverse populations will further help translate how our genome shapes our health to derive treatment options for diseases.

## Methods

### Contributing studies and metabolite measurements

#### Study description

Samples from two UK-based cohort studies—EPIC-Norfolk and INTERVAL—were included in the current analyses^6,7^.

#### INTERVAL

The INTERVAL study (https://www.intervalstudy.org.uk) comprises up to 50,000 participants nested within a randomized trial of varying blood donation intervals recruited at 25 centers of England’s National Health Service Blood and Transplant (NHSBT). All INTERVAL participants gave informed consent before joining the study, and the National Research Ethics Service approved the study (11/EE/0538). INTERVAL participants were not compensated for participation. Participants completed an online questionnaire, including questions about demographic characteristics (for example, age, sex and ethnicity), anthropometry (height and weight), lifestyle (for example, alcohol and tobacco consumption) and diet. Participants were non-fasting and generally in good health, because blood donation criteria exclude people with a history of major diseases (such as myocardial infarction, stroke, cancer, human immunodeficiency virus and hepatitis B or C) and those who have had recent illness or infection. INTERVAL blood samples were taken at baseline, and ethylenediaminetetraacetic acid plasma was stored at −80 °C.

#### EPIC-Norfolk

The EPIC-Norfolk study (https://www.epic-norfolk.org.uk) is a population-based prospective cohort study, nested within the European Prospective Investigation of Cancer (EPIC) study, which had the primary aim of exploring the connections between cancer, diet and lifestyle. EPIC-Norfolk recruited 30,446 men or women aged between 40 and 79 years at baseline, from NHS GP practices in Norfolk, UK, between 1994 and 1997. At baseline, information on diet, lifestyle and self-reported previous diagnosis of disease were collected, and 25,639 participants attended a clinic examination to take blood samples and anthropometric measures. The EPIC-Norfolk study was approved by the Norwich Local Ethics Committee (previously known as Norwich District Ethics Committee) (REC ref: 98CN01); all participants gave their informed written consent before entering the study. Participants did not receive any compensation for their involvement in the EPIC-Norfolk study.

Participants of both studies were generally non-fasting at the time of blood sampling. INTERVAL participants were non-fasting blood donors and EPIC-Norfolk participants were not specifically requested to fast (4.4% of EPIC-Norfolk participants were fasted (≥6 h since last meal)).

A total of 19,994 individuals of European ancestry contributed to the current analysis; 14,296 individuals for discovery (5,841 EPIC-Norfolk samples and 8,455 INTERVAL samples) and 5,698 individuals (from EPIC-Norfolk) for validation (Supplementary Table 1). The mean age in the discovery sample was 59.8 years in the EPIC-Norfolk study and 44.0 years in the INTERVAL study, with 53.3% females in EPIC-Norfolk and 48.8% in INTERVAL.

#### Non-targeted metabolomics

Plasma metabolites were measured using the untargeted DiscoveryHD4 platform (Metabolon), which uses ultra-high-performance liquid chromatography/tandem accurate mass spectrometry and references to a library of biochemicals of known and unknown identity based on standards with mass-to-charge ratio (*m*/*z*), retention time/index and chromatographic data. An in-depth description of the process is described in ref. ^8^. Metabolites were classified by Metabolon into eight broad named classes relating to the metabolism of lipids, amino acids, xenobiotics, nucleotides, peptides, carbohydrates, cofactors and vitamins, and ‘energy’. In addition, there are compounds with undetermined chemical identity (unannotated compounds). The unannotated compounds represent recurring biological entities that have been detected over time across many different studies completed at Metabolon, which has allowed the assignment of these features as unique metabolites despite the lack of full structural elucidation. The process used by Metabolon to associate features relating to the same compound into one library entry has previously been described^61^. In addition, analysis of the various feature types is described in ref. ^62^.

Measurements were made independently in EPIC-Norfolk and INTERVAL. Metabolite values were natural-log-transformed, winsorized to 5 s.d. from the mean, residuals were calculated from a multivariable linear regression model adjusting for age and sex (measurement batch and study-specific variables), and the residuals were standardized (mean = 0, s.d. = 1). Following quality control (QC), 913 metabolites were present in at least 200 participants within the discovery. For INTERVAL, two sub-cohorts of 4,316 and 4,637 participants were created through random sampling from the INTERVAL study and metabolites were measured within these two sub-cohorts (or batches) separately. Within each batch, sample-specific metabolite values were median-normalized for run day (median set to 1 for run-day batch) and imputed (‘ScaledImpData’) by Metabolon. These imputed values were identified using the raw data (‘OrigScale’) provided by Metabolon and were reset to missing prior to QC. Metabolites were then excluded if measured in only one batch or in fewer than 100 samples. We did not observe any technical variability between the batches, so the batches were merged prior to the QC and genetic analysis including batch as a covariate to adjust for any residual batch effects. Metabolite values were natural-log-transformed, then winsorized to 5 s.d. from the mean where the values exceeded mean ± 5 × s.d. of the metabolite. Residuals were then calculated, adjusting for age, sex (self-reported), Metabolon batch, INTERVAL center, plate number, appointment month, the lag time between the blood donation appointment and sample processing, and the first five ancestry principal components. Before the genetic analysis, these residuals were standardized to a mean of 0 and s.d. of 1. For EPIC-Norfolk, untargeted metabolomics measurements were made in 2015–2017, separately in three batches, using the DiscoveryHD4 platform (Metabolon). Citrated plasma samples were stored in the gas phase of liquid nitrogen at −175 °C for long-term storage. Samples were transferred to short-term storage at −70 °C and shipped on dry ice to Metabolon. Initially, metabolites were measured in a diabetes case cohort (*N* = 1,503); for the present analysis we consider only the sub-cohort of the case cohort (*N* = 857). Subsequently two sets of ~6,000 samples were measured (*N* = 5,994 and *N* = 6,173; the latter including almost 200 duplicates), which were quasi-random selections. Due to the timing of measurements, EPIC-Norfolk samples were divided into a set to contribute to the ‘discovery set’ and a separate ‘validation set’. The combined sub-cohort and first quasi-random selection were treated as the EPIC-Norfolk constituent of the ‘discovery set’ and the second quasi-random selection constituted the ‘validation set’. Following the exclusion of duplicated samples, samples from participants withdrawn from the study, samples without genotype data passing QC, the total numbers of EPIC-Norfolk individuals in the discovery and validation sets were 5,841 and 5,698, respectively. For the case cohort, metabolite levels scaled to set the median equal to 1, provided by Metabolon, were used. For the two sets of ~6,000 samples, values were additionally normalized by the volume extracted before being scaled to set the median equal to 1. Imputed values were not used. Metabolites were excluded if measured in fewer than 100 samples in the respective discovery/validation set. Within the measurement batch, among genotyped samples, metabolite values were natural-log-transformed and winsorized to 5 s.d. from the mean (using Stata 14.2). Within the discovery/validation set, samples without genotype data passing QC were excluded, residuals were calculated using linear regression adjusting for age and sex (self-reported but participants with sex chromosomes discordant from self-reported sex were excluded) (and measurement batch), and the residuals were standardized to a mean of 0 and s.d. of 1 (using R version 3.2.2).

Metabolites were matched between studies based on the Metabolon ‘chemical id’ where present or names in the case of unannotated metabolites. A complete list of metabolites, chemical IDs (CHEMICAL_ID) and compound IDs (COMP_ID) for each constituent measurement batch are given in Supplementary Table 2.

### Genotyping and imputation

The genotyping protocol and QC for the INTERVAL samples (up to 50,000) have been described previously in detail^6^. In short, DNA extracted from buffy coat was used to assay ~830,000 variants on the Affymetrix Axiom UK Biobank genotyping array at Affymetrix. Genotyping was performed in multiple batches of ~4,800 samples each, and sample QC was performed, including exclusions for sex mismatches, low call rates, duplicate samples, extreme heterozygosity and non-European descent. Multidimensional scaling was performed using PLINK v1.9 to create components to account for ancestry in genetic analyses. Before imputation, additional variant filtering steps were performed to establish a high-quality imputation scaffold. In summary, 654,966 high-quality variants (autosomal, non-monomorphic, bi-allelic variants with Hardy–Weinberg equilibrium (HWE) *P* > 5 × 10^−6^, with a call rate of >99% across the INTERVAL genotyping batches in which a variant passed QC, and a global call rate of >75% across all INTERVAL genotyping batches) were used for imputation. Variants were phased using SHAPEIT3 and imputed using a combined 1000 Genomes Phase 3-UK10K reference panel. Imputation was performed via the Sanger Imputation Server (https://imputation.sanger.ac.uk) and resulted in 87,696,888 imputed variants. Variants with MAF < 0.01% or INFO (imputation INFO score) of <0.3 were excluded before further analysis. For EPIC-Norfolk, samples (*N* = 21,448) were genotyped on the Affymetrix UK Biobank Axiom array chip by Cambridge Genomic Services. Sample and variant QC followed the Affymetrix Best Practices guidelines. Samples were excluded based on DishQC < 0.82 (fluorescence signal contrast), call rate of <97%, heterozygosity outliers and sex discordance checks. Variants were excluded if the call rate was <95% or HWE *P* ≤ 1 × 10^−6^. Monomorphic variants and those with cluster problems detected using Affymetrix SNPolisher were excluded. Genotype imputation was performed using two different reference panels, the Haplotype Reference Consortium (HRC) (release 1) reference panel and the combined UK10K + 1000 Genomes Phase 3 reference panel. After pre-imputation QC, 21,044 samples remained for imputation. All variants imputed using the HRC reference panel were included, and additional variants imputed using only the UK10K + 1000 Genomes reference panel were added to create a combined imputed set. Variants with imputation quality INFO < 0.4 or minor allele count (MAC) of ≤2 were excluded. All positions were on genome assembly GRCh37.

### Discovery GWAS and meta-analysis

GWASs were performed separately in INTERVAL (*N* = 8,455) and EPIC-Norfolk (*N* = 5,841), for each metabolite using BOLT-LMM^63^ (version 2.2). Where BOLT-LMM failed, for example, due to an invalid heritability estimate close to 0 or 1, the analysis was run using SNPTEST (version 2.5.1 or 2.5.2)^64,65^. In SNPTEST analyses, related individuals were excluded and the first genetic principal components (five for INTERVAL and four for EPIC-Norfolk) were included. Variants with MAF < 0.01%, imputation quality INFO < 0.3, HWE *P* < 1 × 10^−6^ or exact alleles unknown, and associations with absolute (effect) >10 or standard error <0 or >10 were excluded. In INTERVAL, for variants with both imputed and genotyped data, imputed data were used if the INFO score was greater than 0.6; otherwise, genotyped data were used. Study-specific results were pooled using inverse-variance weighted fixed-effect meta-analyses and METAL^66^, applying a MAC threshold of >10 in each study.

### Definition of genomic regions

To define regions, all associations with *P* < 5 × 10^−8^ in the meta-analysis and *P* < 0.01, MAC > 10 and consistent direction of effect in both studies were taken forward. Pairwise LD was calculated within the INTERVAL study (*N* ≈ 50,000). For each individual metabolite, sentinel variants (with the largest −1*log10(*P*)) were identified and the range of positions of variants in LD (*r*^2^ ≥ 0.1) was used to define the region. For sentinel variants with no other variants in LD, a region around the sentinel variant (±500 kb) was created. In the next step, sentinel variants for all metabolites were considered. Pairwise LD was calculated for each sentinel variant, and regions with sentinel variants in LD (*r*^2^ > 0.6) were merged and a further 250 kb was added to either side of each region to avoid having variants in the margin of the locus. Overlapping regions were merged until all defined regions were non-overlapping.

### Validation of metabolite–region associations

We validated regional sentinel variant–metabolite associations by meta-analyzing the discovery and validation data. The GWAS for the validation data was performed using the same protocol as for the discovery data. Associations were considered validated if the association was significant after correction for multiple testing (*P* < 5.48 × 10^−11^) in the validation meta-analysis, with consistent direction of effect in all three constituent GWASs.

### Conditional analysis

Exact conditional analysis was performed using combined individual-level data from INTERVAL and EPIC-Norfolk discovery sets. We performed forward stepwise regression analyses, adjusting for fixed effects of the study and the top genetic principal components and considering variants with consistent direction of effect and *P* < 0.01 in both discovery datasets. Region-wide association analyses were performed using SNPTEST (version 2.5.2). Initially, we conditioned on the most strongly associated regional variant from marginal analyses and estimated the association of each other regional variant independently in the conditional model. We then identified the variant with the lowest *P* value from the tested regional variants, added it to the conditional model, and re-estimated associations of all other regional variants using the updated conditional model. This process was repeated iteratively until no further regional variants were significant at *P* < 1.25 × 10^−8^. The *P* < 1.25 × 10^−8^ threshold was calculated using the Bonferroni correction, adjusting for the maximum number of variants (*n* = 39,297) and metabolites (*n* = 102) tested at any region. We fitted a final linear regression model (R version 3.2.2), and excluded any selected variants not significant at *P* < 1.25 × 10^−8^ in the full conditional model. In a small number of instances (*n* = 49; 2.65%), no regional variant, including the lead variant, associated at *P* < 1.25 × 10^−8^ (which was more stringent than the discovery analysis threshold of *P* < 5 × 10^−8^). In this situation we ran conditional analyses conditioning on the lead variant and, if no other variants were found to be associated at *P* < 1.25 × 10^−8^, we retained only the original lead variant.

### Technical validation of rare variant associations

To ensure that rare variant associations were not due to technical artefacts of the imputation, we performed a technical validation using WES data in a subset of 3,924 samples from the INTERVAL study^9^. We looked up associations from analysis of the INTERVAL WES data for 122 (49.8%) of the total 245 rare variant associations for which variants and metabolites overlapped. All associations were directionally consistent with our analysis with an almost perfect correlation of effects (*r*^2^ = 98.33), and 118 were at least nominally significant (*P* < 0.05) (Extended Data Fig. 2).

### Definition of GIMs

A matrix (‘matrix.ref’) was created with the variants from the conditional analysis for all regionally associated metabolites as rows, metabolites as columns and −log10(*P*) for conditional association with each metabolite as individual elements of the matrix (Extended Data Fig. 1). The variant with the largest −log10(*P*) for association with any metabolite in ‘matrix.ref’ was selected, and −log10(*P*) for association of the selected variant with all the metabolites within the matrix was calculated and added to a new matrix called ‘matrix.out’. This variant was removed from ‘matrix.ref’ and the −log10(*P*) for the association of each of the remaining variants in the ‘matrix.ref’ was calculated, conditioning on the variant(s) in ‘matrix.out’. The steps were repeated by selecting the next variant with the largest −log10(*P*) within ‘matrix.ref’, adding it to ‘matrix-out’ and estimating the associations of each variant and each metabolite in ‘matrix-ref’, conditioning on all variants in ‘matrix-out’. This was repeated until no variant–metabolite association was identified in ‘matrix.ref’ with a −log10(*P*) > −log10(5 × 10^−8^). Every time we selected the variant with the largest −log10(*P*) for association with any given metabolite within ‘matrix.ref’, we ensured that this variant–metabolite association had the same direction of effect with a *P* value for association with the metabolite of less than 0.01 in both INTERVAL and EPIC-Norfolk. A marker order number was assigned to indicate the order in which variants were included in ‘matrix.out’. In the last step, we created metabotypes within the locus using the genetic associations within ‘matrix.out’ with −log10(*P*) > −log10(5 × 10^−8^). Starting with the first variant, all metabolites with a significant association were selected, then we selected all variants associated with any one of these metabolites. This step was repeated until no variant or metabolite was added to the metabotype.

We reviewed all GIMs manually to check whether adjacent regions with the same metabolites were inadvertently split, and identified ten such regions. These adjacent regions were manually merged, and the GIMs were recalculated within the merged (extended) region. This method of defining GIMs was ‘hypothesis-free’ and inclusive, based on genetic associations. It did not take account of existing biological relationships or phenotypic correlations between metabolites. We examined phenotypic correlations among metabolites within GIMs and include these in Supplementary Table 15.

The −log10(*P*)s in the matrices, along with the +/− that represent the direction of effect (Supplementary Table 7), are for the associations from stepwise selection mentioned above; that is, they are conditional on only the variants with lower marker order numbers (column ‘Marker Order (Conditional Analysis)’), as opposed to all the variants in the GIMs in that region.

### Causal gene annotation

The biochemical investigation of living systems preceded GWAS by many decades. Often, the names of genes and proteins reflect their biochemical activity. We used both these facts to deduce the likely causal genes at many of the metabolite-associated variants. Specifically, we used automated approaches to identify potential supporting information for the 20 closest protein-coding genes to each lead variant, using distance from lead variant to the ‘gene body’ (transcription start site to transcription end site) of each gene. This information was manually reviewed to identify the most likely causal gene for each locus.

To leverage the fact that many gene names directly reflect their known substrates (for example, phenylalanine hydroxylase), we used the following approaches:

1. Fuzzy text match (Ruby Gem fuzzy_match, score > 0.5) of any synonym of the metabolite name (from HMDB) to the name of the gene (entrez) or the name of the protein or a synonym of the name of the protein (UniProt).
2. Fuzzy text match of the class of the metabolite (from HMDB) to the name of the protein (UniProt).
3. Fuzzy text match of any synonym of the metabolite to the names of any rare diseases caused by the gene (OMIM) after removing the following stop words: uria, emia, deficiency, disease, transient, neonatal, hyper, hypo, defect, syndrome, familial, autosomal, dominant, recessive, benign, infantile, hereditary, congenital, early-onset, idiopathic.

To leverage known biochemical pathway knowledge we used the following approaches:

1. Lookup of candidate genes in HMDB’s interacting proteins annotation
2. Match of KEGG maps between each metabolite and each gene (no direct connection required, just co-occurrence on a KEGG map).
3. Fuzzy text match of any synonym of the metabolite to the set of GO biological processes with fewer than 500 human genes to which each gene was assigned after removing the following non-specific substrings from the name of the biological process: metabolic process, metabolism, catabolic process, response to, positive regulation of, negative regulation of, regulation of.

Any positive hits from the above automated analyses were manually reviewed, as well as any supporting primary literature. If the existing experimental evidence convincingly supported one of the 20 genes at the locus, that gene was selected as the biologically most likely causal gene. If there was no clear experimental evidence for any of the 20 closest genes, no causal gene was manually selected. In some cases, two or more genes at a locus had equally strong experimental evidence. This is especially the case with nearby paralogs arising from gene duplication. In these cases, multiple causal genes have been flagged, indicating that one or more of the selected genes may be contributing to the metabolite–variant association.

### Assessing novelty of associations

We assessed the novelty of variant associations using associations reported by the previous two largest genetic studies that used the Metabolon assay^4,5^. Based on a 250-kb-distance-based window, we identified 631 region–metabolite associations reported by these studies. Of these, 83 region–metabolite associations were with ratios and were excluded from the comparison. Of the remaining 548 region–metabolite associations, we identified 302 region–metabolite associations as significant in our study (at *P* < 5 × 10^−8^; Supplementary Table 6). Within the remaining 246 region–metabolite associations, for 185 region–metabolite associations we were not able to map the metabolite to our study. Therefore, of the 363 region–metabolite associations (involving 118 genomic regions and 243 metabolites) where metabolites were directly mapped to our study, we replicated (at *P* < 5 × 10^−8^; associations with either the reported variant or a variant in LD, *r*^2^ > 0.1) 302 (83.2%) of the region–metabolite associations (involving 106 genomic regions and 226 metabolites).

### Colocalization

Where we report results from colocalization analyses, the analyses were performed using HyPrColoc^18^. In the first step we performed pairwise colocalization to derive the list of metabolites that colocalize with the outcome. The selected set of metabolites were then colocalized using the multi-trait colocalization framework described in the HyPrColoc manuscript^18^. We report only the colocalizations with a PP greater than 0.8. The following datasets were used in the colocalization analysis: gene expression (GTEx Analysis Release V8)^28^, breast cancer^42^ and depression^19^.

### Enrichment of genes known to cause IEMs

Genes were mapped to metabolic regions using two methods: (1) manual annotation of likely causal genes based on the biochemical literature as previously described and (2) a gene set consisting of the closest gene to any conditionally independent variant. For method (2), the closest gene was identified using Variant Effect Predictor (VEP), and genes within 5 kb of conditionally independent variants and their proxies (*r*^2^ > 0.6) were identified using SNiPA. These genes were assessed for known causal links to IEMs using a list of 785 known IEM genes downloaded from the Orphanet database^67^.

We tested whether there was enrichment of IEM genes among all genes annotated to metabolite-associated regions compared to the percentage of known IEM-linked genes^67^ (*n* = 785; 4%) among genome-wide protein-coding genes (*N* = 19,817)^68^, using a two-tailed binomial test. As a sensitivity analysis, enrichment was assessed using less specific methods of assigning genes to metabolic regions, where genes were identified within 500 kb of conditionally independent variants or within the genomic region using GENCODE. Supplementary Table 16 provides a summary and comparison of these methods.

### Phenotypic assessment of metabolite-associated variants at the *DBH* and *TH* loci

Complex phenotype associations reported in GWAS Catalog, PhenoScanner^40^ and UK Biobank at a significance threshold of *P* = 1 × 10^−5^ were identified for rs6271 at the *DBH* locus, rs10840516 at the *TH* locus, and any variants that were in strong LD (*r*^2^ ≥ 0.8). Associations for which the phenotypes were related to one or more symptoms of the corresponding IEMs, orthostatic hypotension (OMIM #223360) and Segawa syndrome (OMIM #605407), as reported in IEMBase and other relevant literature, were tested for colocalization with metabolite levels. A list of associations at *DBH* and *TH* loci that were prioritized is provided in Supplementary Table 17. To test for colocalization between variant–metabolite associations and variant–phenotype associations, multi-trait colocalization was implemented using the R package ‘HyPrColoc’ (v1.0)^18^. To maximize statistical power, summary statistics from UK Biobank were used for phenotypes relating to blood pressure, hypertension, body fat composition and medication, and summary statistics from the SSGAC consortium were used for ‘Years of schooling’ (Supplementary Table 17). The prior probability that a variant is associated with a single trait (prior1) was set to 1 × 10^−4^, and the prior probability that a variant is associated with one trait, given it is already associated with another (prior2), was set to 0.98. Regional and alignment probability thresholds were set to 0.5. Cluster stability was assessed by using more stringent prior2 values (0.99, 0.999) and regional and alignment threshold values (0.6, 0.7, 0.8, 0.9). Only variants present in all included traits were considered for a given locus and any variants with a standard error of zero were removed.

### GIMs enable variant-to-function annotation at GWAS loci

To identify complex diseases associated with the sentinel variants (or proxies at *r*^2^ > 0.8) for our 423 GIMs, we queried the NHGRI-EBI GWAS catalog and other GWAS cataloged within PhenoScanner^40,69^. A total of 97 phenotypes from the GWAS catalog were then manually classified into 52 disease categories with EFO terms before investigating the LD between the GIM variants and top disease variant and further colocalization for specific selected associations using HyPrColoc^18^. To assess whether the causal genes are druggable, we looked at whether the genes are either targets of approved small molecules and biotherapeutic drugs (Tier 1) or clinical-phase drug candidates or encode targets with known bioactive drug-like small-molecule binding partners as well as those with ≥50% identity (over ≥75% of the sequence) with approved drug targets (Tier 2), as reported in ref. ^41^.

### Phenome-wide associations of metabolite levels

To facilitate phenome-association studies for metabolites, we imputed plasma metabolite levels in UK Biobank participants using conditionally independent metabolite-associated variants (Supplementary Table 4) with exact variant mappings. We created weighted (by the marginal effect) summed scores of the genetic load for metabolite levels for each of 155 metabolites with at least two variants and a clear metabolite annotation (using Stata 14.0 and R 3.6.0). We included only variants associated (*P* < 5 × 10^−8^ in the marginal statistics) with fewer than five metabolites to minimize the impact of horizontal pleiotropy. We used these genetic scores as exposure variables, testing for associations with 1,457 phecodes, adjusting for age, sex (reported, but participants with sex chromosomes discordant from reported sex were excluded), genotype batch, test center and the first ten genetic principal components. We performed logistic regression models (using R 3.6.0) within up to 351,967 unrelated participants of white European ancestry^70^. To generate phecode-based outcome variables, we mapped ICD-10, ICD-9, Read version 2 and Clinical Terms Version 3 (CTV3) terms from self-report or medical health records, including cancer registry, death registry, hospitalization (Hospital Episode Statistics) and primary care (subset, *N* = 214,667), to the phecodes^45,71^. We used any code that was recorded, irrespective of whether it contributed to the primary cause of death or hospital admission, to define phecodes. We adjusted all analyses for test center to account for regional differences in coding systems and case ascertainment. For each participant and phecode, we kept only the first entry, irrespective of the original dataset, generating a first occurrence dataset. We dropped codes that were before or in the participants’ birth year to minimize coding errors from electronic health records. To account for multiple testing, we applied the Benjamin–Hochberg procedure to the full list of genetic score to phecode associations tested, controlling the false discovery rate at 5%. To test whether single variants rather than the genetic score for a metabolite accounted for the observed associations, we repeated the same analysis for single variants only, and flagged all examples for which the strongest single variant was more strongly associated with the phecode compared to the composite score. To further test for a dose–response relationship, we adapted a two-sample MR framework^72^. We used heterogeneity estimates from an inverse-variance weighted MR along with MR-Egger to test for horizontal pleiotropy, and Cochran’s Q statistic to test for heterogeneity among effect estimate ratios for each variant included.

### Reconstruction of the metabolic network using Gaussian graphical models

We imputed missing values for 749 metabolites with fewer than 30% missing observations in each of the INTERVAL and EPIC-Norfolk discovery datasets, individually within the study. Missing observations were imputed using multivariate imputation by chained equations (MICE), implemented using R (3.3.3) and the R package ‘mice’ (version 2.46.0)^73^ with the method ‘norm’, as previously proposed for metabolomics data^74^. Imputation was performed on the residuals after taking metabolite measures that were median-normalized for assay run day, natural-log-transformed, winsorized to 5 s.d. and regressing out the effects of age, sex and study-specific covariates. The imputation model for each metabolite considered other metabolites (with fewer than 30% missing values). Thirty multiple imputations were performed, each with 50 iterations of the chain. This procedure reasonably assumes that the missing metabolite values can be explained by the values and relationships between the observed metabolite values, but are independent of the unobserved metabolite values.

To construct a data-derived metabolic network^75^, we estimated partial correlations between metabolites in the following manner. Within each study, for each of the 30 imputed datasets, imputed measures were standardized (mean = 0, s.d. = 1) and used to estimate partial correlations between metabolites with the R package ‘GeneNet’^76^ (version 1.2.13). Partial correlation estimates were transformed using Fisher’s *z* transformation with the R package ‘psych’^77^ (version 1.7.8), and estimates for the 30 sets were pooled within the study using Rubin’s rules^78^. The pooled estimates for the two studies were meta-analyzed using a fixed-effect, inverse-variance weighted method implemented using the R package ‘meta’^79^ (version 4.3-0) and back-transformed to correlation estimates.

The Gaussian graphical models (GGMs) resulting from inclusion of absolute partial correlations greater than 0.10, 0.12 or 0.15 can be viewed at http://omicscience.org/apps/mgwas. In the networks, nodes (circles) represent metabolites and black edges the partial correlations between metabolites. Solid lines indicate positive partial correlations and dashed lines negative partial correlations. To the GGMs we added the GWAS results by connecting candidate genes (gray squares) to metabolites (green edges). Candidate genes were from two sources: (1) ‘From literature’, which are those annotated as the causal gene for a GIM in which the metabolite lies (as described in the section ‘Causal gene annotation’) and (2) ‘SNiPA / VeP’, which are genes annotated to the variants defining a GIM in which the metabolite lies by SNiPA/VeP. We used a systems biology approach to annotate compounds based on their metabolic neighborhood and genetic associations in the generated network to enable prediction of pathway membership and chemical identity for unannotated metabolites present in the imputed dataset (*n* = 224)^80^ (Supplementary Tables 18 and 19).

### Reporting summary

Further information on research design is available in the Nature Research Reporting Summary linked to this Article.

## Online content

Any methods, additional references, Nature Research reporting summaries, source data, extended data, supplementary information, acknowledgements, peer review information; details of author contributions and competing interests; and statements of data and code availability are available at 10.1038/s41591-022-02046-0.

## Supplementary information

Reporting Summary

Supplementary Tables Supplementary tables.

## Data Availability

We provide open access to all summary statistics for academic use through an interactive webserver: https://omicscience.org/apps/mgwas. Metabolite raw relative abundances are available at https://www.ebi.ac.uk/metabolights/ (project codes: MTBLS833 and MTBLS834). The EPIC-Norfolk data can be requested by bona fide researchers for specified scientific purposes via the study website (https://www.mrc-epid.cam.ac.uk/research/studies/epic-norfolk/). Data will either be shared through an institutional data sharing agreement or arrangements will be made for analyses to be conducted remotely without the need for data transfer. INTERVAL study data from this paper are available to bona fide researchers from helpdesk@intervalstudy.org.uk and information, including the data access policy, are available at http://www.donorhealth-btru.nihr.ac.uk/project/bioresource.

## Code availability

Code used for analysis in this study is available on GitHub (https://github.com/MRC-Epid/MetabolomicsGWAS_INTERVAL_EPICNorfolk).
