# Supplementary material for: Rare and common genetic determinants of metabolic individuality and their effects on human health
Source: Nat Med. 2022 Nov 10;28(11):2321–32. doi: 10.1038/s41591-022-02046-0 (PMC9671801; doi:10.1038/s41591-022-02046-0)
Supplement: Supplementary file 1 — Reporting Summary [file 41591_2022_2046_MOESM1_ESM.pdf]

Corresponding author(s): Claudia Langenberg
Last updated by author(s): 13/09/2022

Reporting Summary

Nature Portfolio wishes to improve the reproducibility of the work that we publish. This form provides structure for consistency and transparency in reporting. For further information on Nature Portfolio policies, see our Editorial Policies and the Editorial Policy Checklist.

Statistics

For all statistical analyses, confirm that the following items are present in the figure legend, table legend, main text, or Methods section.

n/a	Confirmed	
☐	☒	The exact sample size (*n*) for each experimental group/condition, given as a discrete number and unit of measurement
☐	☒	A statement on whether measurements were taken from distinct samples or whether the same sample was measured repeatedly
☐	☒	The statistical test(s) used AND whether they are one- or two-sided<br>*Only common tests should be described solely by name; describe more complex techniques in the Methods section.*
☐	☒	A description of all covariates tested
☐	☒	A description of any assumptions or corrections, such as tests of normality and adjustment for multiple comparisons
☐	☒	A full description of the statistical parameters including central tendency (e.g. means) or other basic estimates (e.g. regression coefficient) AND variation (e.g. standard deviation) or associated estimates of uncertainty (e.g. confidence intervals)
☐	☒	For null hypothesis testing, the test statistic (e.g. *F*, *t*, *r*) with confidence intervals, effect sizes, degrees of freedom and *P* value noted<br>*Give P values as exact values whenever suitable.*
☒	☐	For Bayesian analysis, information on the choice of priors and Markov chain Monte Carlo settings
☒	☐	For hierarchical and complex designs, identification of the appropriate level for tests and full reporting of outcomes
☐	☒	Estimates of effect sizes (e.g. Cohen's *d*, Pearson's *r*), indicating how they were calculated

Our web collection on statistics for biologists contains articles on many of the points above.

Software and code

Policy information about availability of computer code

Data collection	The INTERVAL study genotype and metabolomics data were QCd and processed at Cardiovascular Epidemilogy Unit, Department of Public Health and Primary Care, University of Cambridge. All preparation and analysis of data from the EPIC-Norfolk cohort were done at the MRC Epidemiology Unit, Cambridge, UK.
Data analysis	We used open source software and programs to perform all analyses. Specific details of the program/software used, including versions, are provided within the methods and supplementary information.<br>Code used for analysis in this study is available on GitHub (https://github.com/MRC-Epid/MetabolomicsGWAS_INTERVAL_EPICNorfolk)<br>The following programs were used to perform various analysis as explained in the manuscript: BOLT-LMM (2.2), SNPTEST (2.5.1,2.5.2), R (3.2.2, 3.3.3, 3.6.0), STATA (14.0, 14.2), METAL (released on 25/03/2011), HyPrColoc (v1.0), Phenoscanner (V2)

For manuscripts utilizing custom algorithms or software that are central to the research but not yet described in published literature, software must be made available to editors and reviewers. We strongly encourage code deposition in a community repository (e.g. GitHub). See the Nature Portfolio guidelines for submitting code & software for further information.

Data

Policy information about availability of data

All manuscripts must include a data availability statement. This statement should provide the following information, where applicable:

- Accession codes, unique identifiers, or web links for publicly available datasets
- A description of any restrictions on data availability
- For clinical datasets or third party data, please ensure that the statement adheres to our policy

We provide open access to all summary statistics for academic use through an interactive webserver: https://omicscience.org/apps/mgwas. Metabolite raw relative

abundances are available at,

https://www.ebi.ac.uk/metabolights/MTBLS833/descriptors
https://www.ebi.ac.uk/metabolights/MTBLS834/descriptors

The EPIC-Norfolk data can be requested by bona fide researchers for specified scientific purposes via the study website (https://www.mrc-epid.cam.ac.uk/research/studies/epic-norfolk/). Data will either be shared through an institutional data sharing agreement or arrangements will be made for analyses to be conducted remotely without the need for data transfer.

INTERVAL study data from this paper are available to bona fide researchers from helpdesk@intervalstudy.org.uk and information, including the Data Access Policy, can be found here: http://www.donorhealth-btru.nihr.ac.uk/project/bioresource

The following data resources were used: Ensembl Variant Effect Predictor (VEP), ClinVar, Orphanet, Online Mendelian Inheritance in Man (OMIM), GTEX (V8), GENCODE

Field-specific reporting

Please select the one below that is the best fit for your research. If you are not sure, read the appropriate sections before making your selection.

☒ Life sciences ☐ Behavioural & social sciences ☐ Ecological, evolutionary & environmental sciences

For a reference copy of the document with all sections, see nature.com/documents/nr-reporting-summary-flat.pdf

Life sciences study design

All studies must disclose on these points even when the disclosure is negative.

Sample size

We included a total of 19,994 participants from the INTERVAL and EPIC-Norfolk studies with both genotype and metabolomics data available. 14,296 participants were included in the discovery analysis (INTERVAL N=8,455; EPIC-Norfolk N=5,841) and an additional 5,698 participants (from EPIC-Norfolk) were included in the validation meta-analysis.

Data exclusions

INTERVAL: Two sub cohorts of 4,316 and 4,637 participants were created through random sampling from the INTERVAL study and metabolites were measured within these two sub cohorts (or batches) separately. Metabolites were then excluded if measured in only one batch or in less than 100 samples. Genotyping protocol and QC for the INTERVAL samples including sample exclusion (up to 50,000) have been described previously in detail (reference below).
Sun, B. B. et al. Genomic atlas of the human plasma proteome. Nature (2018) doi:10.1038/s41586-018-0175-2.

EPIC-Norfolk: Untargeted metabolomics measurements were made in 2015-2017, separately in three batches, using the DiscoveryHD4® platform (Metabolon, Inc., Durham, USA). Initially metabolites were measured in a diabetes case-cohort (N=1,503). Subsequently two sets of ~6000 samples were measured (N=5,994 and N=6,173; the latter including almost 200 duplicates). From the case-cohort, we excluded samples (n=646) not in the sub-cohort. We excluded duplicated samples, samples from participants withdrawn from the study and samples without genotype data passing quality control.

Replication

In the absence of a similarly powered external replication dataset, we performed a two stage meta-analyses to discover and validate genetic associations with metabolites. In the first stage, top hits (n=3271) for each metabolite were selected at P-value<5e-08. In the second stage, meta-analyses was performed including the discovery studies and an additional 5,698 participants from the EPIC-Norfolk study. Following this analysis, 1847 variants were deemed significant at P-value<1.25e-11 in this meta-analyses and were followed up (Supplementary Table 3).

Randomization

EPIC-Norfolk participants were selected for metabolomics measurements in 2 stages; first a diabetes case-cohort study (N=1,503; sub-cohort n=857) of incident cases and a randomly drawn sub-cohort, second a sub-cohort of eligible participants was drawn in a quasi-random manner and measured in 2 batches.

Blinding

Blinding was not required. The analysis was performed using continuous metabolite data, and no case/control status were used.

Reporting for specific materials, systems and methods

We require information from authors about some types of materials, experimental systems and methods used in many studies. Here, indicate whether each material, system or method listed is relevant to your study. If you are not sure if a list item applies to your research, read the appropriate section before selecting a response.

Materials & experimental systems

n/a	Involved in the study
☒	☐ Antibodies
☒	☐ Eukaryotic cell lines
☒	☐ Palaeontology and archaeology
☒	☐ Animals and other organisms
☐	☒ Human research participants
☒	☐ Clinical data
☒	☐ Dual use research of concern

Methods

n/a	Involved in the study
☒	☐ ChIP-seq
☒	☐ Flow cytometry
☒	☐ MRI-based neuroimaging

Human research participants

Policy information about studies involving human research participants

Population characteristics	INTERVAL study participants included in this study were healthy blood donors. Mean age 44, 49% were males and of European ancestry where the self reported sex was used.<br>EPIC-Norfolk is a population based cohort from Norfolk in Eastern England. Participants included in this study were on average 60 years old and 53% were female where the sex was self-reported. Participants with sex chromosomes discordant from self-reported sex were excluded.<br>A more detailed description is given in Supplemental Table S1.
Recruitment	The INTERVAL study comprises up to 50,000 participants nested within a randomized trial of varying blood donation intervals recruited at 25 centres of England's National Health Service Blood and Transplant (NHSBT).<br>EPIC-Norfolk (https://www.epic-norfolk.org.uk/; PMID: 10466767): is a population-based prospective cohort study, nested within the European Prospective Investigation of Cancer (EPIC). EPIC-Norfolk recruited 30,446 men or women aged between 40 and 79 years at baseline, from NHS GP practices in Norfolk, UK, between 1994 and 1997.
Ethics oversight	All INTERVAL participants gave informed consent before joining the study and the National Research Ethics Service approved this study (11/EE/0538). INTERVAL participants were not compensated for participation.<br>The EPIC-Norfolk study was approved by the Norwich Local Ethics Committee (previously known as Norwich District Ethics Committee) (REC Ref: 98CN01); all participants gave their informed written consent before entering the study. Participants did not receive any compensation for their involvement in the EPIC-Norfolk study.

Note that full information on the approval of the study protocol must also be provided in the manuscript.
